# One Step Forward, Two Steps Back; Xeno-MicroRNAs Reported in Breast Milk Are Artifacts

**DOI:** 10.1371/journal.pone.0145065

**Published:** 2016-01-29

**Authors:** Caner Bağcı, Jens Allmer

**Affiliations:** 1 Department of Biotechnology, Izmir Institute of Technology, Urla, Izmir, Turkey; 2 Department of Molecular Biology and Genetics, Izmir Institute of Technology, Urla, Izmir, Turkey; 3 Bionia Incorporated, IZTEKGEB A8, Urla, Izmir, Turkey; CSIR Institute of Genomics and Integrative Biology, INDIA

## Abstract

**Background:**

MicroRNAs (miRNAs) are short RNA sequences that guide post-transcriptional regulation of gene expression via complementarity to their target mRNAs. Discovered only recently, miRNAs have drawn a lot of attention. Multiple protein complexes interact to first cleave a hairpin from nascent RNA, export it into the cytosol, trim its loop, and incorporate it into the RISC complex which is important for binding its target mRNA. This process works within one cell, but circulating miRNAs have been described suggesting a role in cell-cell communication.

**Motivation:**

Viruses and intracellular parasites like *Toxoplasma gondii* use miRNAs to manipulate host gene expression from within the cellular environment. However, recent research has claimed that a rice miRNA may regulate human gene expression. Despite ongoing debates about these findings and general reluctance to accept them, a recent report claimed that foodborne plant miRNAs pass through the digestive tract, travel through blood to be incorporated by alveolar cells excreting milk. The miRNAs are then said to have some immune-related function in the newborn.

**Principal Findings:**

We acquired the data that supports their claim and performed further analyses. In addition to the reported miRNAs, we were able to detect almost complete mRNAs and found that the foreign RNA expression profiles among samples are exceedingly similar. Inspecting the source of the data helped understand how RNAs could contaminate the samples.

**Conclusion:**

Viewing these findings in context with the difficulties foreign RNAs face on their route into breast milk and the fact that many identified foodborne miRNAs are not from actual food sources, we can conclude beyond reasonable doubt that the original claims and evidence presented may be due to artifacts. We report that the study claiming their existence is more likely to have detected RNA contamination than miRNAs.

## Introduction

Mature microRNAs (miRNAs) are short RNAs (~22 nt) that guide post-transcriptional gene regulation by base pairing with their target mRNAs. They were first discovered in *Caenorhabditis elegans* [1] and have since attracted increasing attention. MicroRNAs have been found in species ranging from viruses to humans [2,3]. Mature miRNAs derive from a stem loop structure called pre-miRNA which, in turn, is cleaved from a pri-miRNA by Drosha (metazoan; DCL1 for plants). The mature miRNA is produced by Dicer and RISC which incorporates one sequence into its complex to guide recognition of target mRNAs. More information about this process can be found in recent reviews [4,5]. MicroRNAs are important regulators of gene expression and their dysregulation may lead to disease [6]. It has been established that viruses encode miRNAs that can regulate host gene expression [7]. It is obviously advantageous for the virus to control the expression of a large array of genes by encoding for a small number of miRNAs. On the other hand, the host may also encode for miRNAs that can target virus mRNAs; or can lose targets for the virus-encoded miRNAs during evolution [8]. Foreign miRNAs, which we will call xeno-miRNAs in the following text, thus, could potentially cause cross-kingdom gene regulation. For viruses this regulation option seems evident and we recently performed a study which shows that intracellular pathogens like *Toxoplasma gondii*, may also employ this type of regulation [9]. According to our computational analysis, *T*. *gondii* may be able to secrete xeno-miRNAs into its host cell to regulate gene expression. Viruses and cell invasive pathogens like *T*. *gondii* can directly interact with the gene expression of their host. For foodborne miRNAs which were first proposed by Zhang et al. [10] such direct interaction is, however, not possible. In this case, the miRNA has to tolerate food processing steps, pass through the digestive tract and the gastrointestinal barrier into the blood before it can reach the cells to regulate gene expression. This finding has been contested multiple times and most recently by RW Lusk [11]. RW Lusk was not able to secure the actual measurements by Zhang et al., but showed experimentally, that finding a foodborne miRNA in plasma is highly unlikely. Around the same time as RW Lusk published his findings, Lukasik and Zielenkiewicz reported the finding of foodborne miRNAs in human and porcine breast milk [12]. The authors took inspiration from the findings of Zhang et al. and analyzed publicly available human and porcine breast milk samples [13,14] which were analyzed using next generation sequencing (NGS) for a different purpose at the Sichuan Agricultural University, Sichuan, China. The authors ignore any contest of the paper by Zhang et al. and report finding large amounts of foodborne miRNAs from multiple plant species; abundantly among them *Arabidopsis thaliana* miRNAs.

Since Arabidopsis is not a food source, this provocative finding inspired the present study, contesting the findings of Lukasik and Zielenkiewicz. We successfully repeated their analyses but then went further and showed that not only miRNAs but also longer transcripts can be found in the samples. Furthermore, the set of transcripts shared among samples (intra- and inter-species) was highly correlated. The chance for such high correlation to occur is extremely low and, therefore, we believe it can much easier be explained through contamination during sample preparation. This notion is further supported by the finding that at the same time when the samples were measured at the Sichuan Agricultural University all species reportedly found to contribute to the miRNAs in breast milk were analyzed at the same institute. Intriguingly, the species with higher number of publications also tend to have higher amount of contamination in the analyzed samples, further supporting this claim.

The most likely conclusion we can draw from our analysis is that the samples measured at the Sichuan Agricultural University were contaminated during the experimental procedure, but after sampling. This finding, in turn, means Lukasik and Zielenkiewicz found RNAs, but that their conclusion that they were xeno-miRNAs performing cross-kingdom regulation was not well supported in the experimental evidence.

## Results and Discussion

### Many of the Previously Reported Plants are not Food Sources

Many miRNAs reported by Lukasik and Zielenkiewicz [12] to be present in breast milk originate from *Arabidopsis thaliana* and it ranked high among the species found in their study (Table 1). Since *A*. *thaliana* is not a food source, we wanted to assess whether the other species that were reported are (Table 1). Table 1 was created by summarizing the supporting information provided by Lukasik and Zielenkiewicz, basically listing the plant species that they identified with their significant identifications for human and pig indicated in the note column (Table 1). We further added 6 animal species as either food sources or negative controls. Excluding our animal additions, 29 plant species’ miRNAs were found 9 of which were not food sources and another 7 which were unlikely. 70% of the plants listed are edible (not necessarily staple foods, though), but at least one is poisonous and another is coated to prevent being eaten. Poplar tree bark for example may be prepared as tea and used for its salicin content, but we doubt that in this manner any detectable amount of RNA will be found in human samples. Using a conservative approach, 60% of the species, whose miRNAs were reportedly detected in breast milk (Table 1), are not food sources for human or pig.

**Table 1:** Plant miRNAs reported to be found in human and/or porcine samples and our assessment of them being food sources. In addition to plant species assessed in the study by Lukasik and Zielenkiewicz, we added some animals (Table 2) indicated as ‘new’. Unlikely food sources are highlighted in bold. X: cannot be tested with the given data. ‘hsa’ and ‘ssc’ in the Note column mean that Lukasik and Zielenkiewicz report a significant identification for human and/or pig, respectively. Data also available as Table A in S2 File.

Popular name	Species	Porcine samples	Human samples	Assessment of being a food source	Note
**Colorado blue Columbine**	***Aquilegia coerulea***		**Found**	**No, all parts are poisonous**	**hsa**
***Arabidopsis lyrata***	***Arabidopsis lyrata***		**Found**	**Leaves are coated to prevent eating**	**hsa**
**Thale cress**	***Arabidopsis thaliana***	**Found**	**Found**	**Not a known food source, but closely related mustard is**	**hsa**
Cow	*Bos taurus*	Found	Found	Yes	new
Purple false brome	*Brachypodium distachyon*		Found	Similar to wheat, but not a major food source	hsa
**Dog**	***Canis familiaris***	**Found**	**Found**	**Not in most parts of the world**	**new**
Citrus	*Citrus paradisi*, *Poncirus trifoliata*		Found	Yes	hsa
Orange	*Citrus sinensis*		Found	Yes	
Citrus	*Citrus trifoliata*	Found	Found	Yes	hsa
**Zebra fish**	***Danio rerio***	**Found**	**Found**	**No**	**new**
Chicken	*Gallus gallus*	Found	Found	Yes	new
Soy bean	*Glycine max*	Found	Found	Yes	hsa, ssc
Soy bean	*Glycine soja*	Found	Found	Yes	hsa
**Human**	***Homo sapiens***	**X**	**Found**	**No**	**new**
Barley	*Hordeum vulgare*	Found	Found	Yes	hsa
**Tobacco**	***Nicotiana tabacum***	**Found**		**No**	
Rice	*Oryza rufipogon*	Found		Yes	
Rice	*Oryza sativa*	Found	Found	Yes	hsa, ssc
**Switchgrass**	***Panicum virgatum***	**Found**		**Food source for herbivores, but not for human or pig**	**ssc**
Runner bean	*Phaseolus coccineus*	Found		Yes	ssc
**Moss**	***Physcomitrella patens***		**Found**	**No, although some are edible**	**hsa**
European spruce tree	*Picea abies*	Found	Found	Maybe a herbal drug, but probably not in Asia	hsa
**Poplar tree**	***Populus euphratica***		**Found**	**No, but used as herbal drug**	
**Poplar tree**	***Populus tremuloides***	**Found**		**No, but used as herbal drug**	**ssc**
**Poplar tree**	***Populus trichocarpa***	**Found**	**Found**	**No, but used as herbal drug**	**hsa, ssc**
Sugarcane	*Saccharum*	Found		Possible food source but not expected in detectable amounts	
Sorghum	*Sorghum bicolor*	Found	Found	Possible food source but not expected in detectable amounts	ssc
Pig	*Sus scrofa*	Found	X	Yes	new
Cacao tree	*Theobroma cacao*		Found	Possible food source but not expected in detectable amounts	
Field penny-cress	*Thlaspi arvense*	Found		Yes, but not expected in detectable amounts	ssc
Bread wheat	*Triticum aestivum*	Found		Yes	ssc
Cowpea	*Vigna unguiculata*	Found	Found	Yes	hsa, ssc
Vine grape	*Vitis vinifera*	Found		Yes, but would it be fed to pigs?	
Corn	*Zea mays*	Found	Found	Yes	hsa, ssc

Other food sources are missing from the table, especially for Sichuan where the experiments were performed, we would expect for example peanut and Chinese cabbage (*Brassica rapa*) miRNAs (both with examples in miRBase [15] and PMRD [16]) to be detected. Human foods like chicken, pig, and other meat sources (many with miRNA examples available on miRBase) are also missing from the results of Lukasik and Zielenkiewicz. While no information about the diet of the human population was provided [13], pigs were apparently fed an undescribed standard feed [14] which probably did not contain several of the plants listed in Table 1 like vine grape, poplar tree, switchgrass, and tobacco. We wanted to investigate whether any meat sources were available in the data and searched for chicken, cow, human, zebrafish, pig, and dog miRNAs. For this aim, we aligned all available mature miRNAs of these species from miRBase to the human and porcine reads allowing no mismatch (Table 2).

**Table 2:** MicroRNAs found in human and porcine samples from selected meat sources. Dog, zebra fish and human were added as controls. X denotes that the assessment is not possible with the given data. Data also available as Table C in S2 File.

Popular name	Species	miRBase	MicroRNAs in human samples	MicroRNAs in porcine samples	Food Source	Total miRNAs in miRBase
**Chicken**	*Gallus gallus*	gga	160	227	Yes	994
**Cow**	*Bos taurus*	bta	273	394	Yes	793
**Pig**	*Sus scrofa*	ssc	229	X	Yes	411
**Dog**	*Canis familiaris*	cfa	203	262	No	453
**Human**	*Homo sapiens*	hsa	X	1062	No	2588
**Zebrafish**	*Danio rerio*	dre	169	209	No	350

We included human, dog and zebrafish as controls and chicken, pig, and cow as examples for major food sources in China where the sampling was performed. The results in Table 2 show significant number of miRNAs mapping to all organisms considered. For pig it could be argued that the evolutionary distance is not too high and similar miRNAs can be retained, but this argument wouldn’t hold for zebrafish or chicken. Additionally, the pig miRNAs found in human samples should somewhat equal the human miRNAs found in pig samples, if they were evolutionary conserved. As this is not the case and since pig samples are generally of lower quality (S1 Fig), we argue that this difference is most likely due to contamination. In summary, many species’ miRNAs were found in breast milk although they are not food sources (~60%) and 100% of the control animal species’ miRNAs were also found in the breast milk samples. This opens the question how they were detected in breast milk samples with a likely explanation being contamination during experimental procedure.

### Route for Foodborne MicroRNAs to be Detectable in Breast Milk

Before we turn to the in-depth analysis of the human and porcine samples, we must recall how breast milk is produced and consider the route foodborne miRNAs have to take to be found in breast milk. This route may encompass:

1. Food production which may involve cooking, baking, fermenting, or many other processing steps which could potentially endanger the structural integrity of small RNAs
2. Passage through the digestive tract unharmed. It is known that at least the duodenum contains nucleases for RNA and DNA
3. Passage from the gastrointestinal barrier into the blood
4. Uptake by alveolar cells
5. Secretion from alveolar cells as milk

These 5 simplified steps contain many different chemical environments with some of them being extremely hostile to small RNAs. Therefore, we believe that if this path is at all possible, a large amount of miRNA must be present in the food source in order to create a measurable titer in breast milk. The miRNA counts, as provided by Lukasik and Zielenkiewicz, which were measured from 20–30 ml breast milk, were, in our opinion, too low (24 clustered reads for the highest abundant miRNA) to be able to significantly affect an infant in any form. We believe that the miRNA titer in a food source must be significantly higher than that because many miRNAs will be degraded along the digestive process. For the transfer into breast milk, it is important to note, that any human cell can uptake the circulating miRNAs from the blood stream, significantly reducing the concentration in the alveolar cells. Finally, the complete contents of these cells are not excreted as milk leading to an additional decrease of the amount of small RNAs detectable therein.

It should, however, be noted that infants do not have a well-developed gastrointestinal barrier thus potentially allowing larger molecules to pass directly into the blood (for example the mother’s antibodies). This would open opportunities for miRNAs to more easily pass into the blood stream if there are any in the milk; perhaps species-specific miRNAs. We conclude that infants might be able to more easily pick-up miRNAs from food, but would not be affected by the small amount of roughly 1 miRNA per ml breast milk as reported. For the transfer of miRNAs from food into breast milk, we conclude that it may only be possible if they are available in excessive amounts, a notion supported by the study of RW Lusk [11]. In the following we will elaborate on why these miRNAs are found in human and porcine breast milk samples.

### Evidence for Messenger RNAs in Human and Porcine Samples

**Fig 1:**
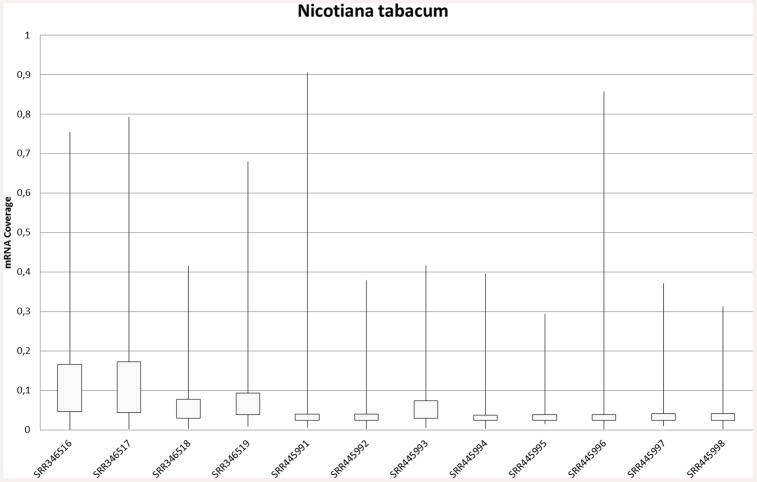
Read Coverage Across Transcripts. Distribution of transcript coverage for *Nicotiana tabacum* in human (first 4) and porcine (last 8) samples. Data can be found in Table N in S4 File.

It can be hypothesized that foodborne miRNAs could be enriched in breast milk since they are rather small. We wondered, however, whether there are only miRNAs in the breast milk samples or whether other RNA species could also be found. Therefore, we mapped reads to available transcripts from *N*. *tabacum* (not a food source), *A*. *thaliana* (unlikely a food source), and *O*. *sativa* (a staple food). First, sequencing adapters were removed from the reads. Then the reads were quality trimmed and aligned to their respective genome (human / pig). For the human samples on average less than 6% of reads remained unaligned, but for the porcine samples on average about 57% of the reads did not align to pig probably mostly due to low quality of sequencing results (compare sequencing quality results in S1 Fig and Table F in S2 File). We retrieved about 3000 transcripts for tobacco from NCBI, ~7000 for *A*. *thaliana* from TAIR, and more than 13000 for rice from PlantGDB (Tables J, N, and R in S3, S4 and S5 Files). In the combined human samples about 200 (~7%) and in the combined porcine samples 6880 (~98%) *A*. *thaliana* transcripts were detected while about 80 were detected in both (Tables K, O, and S in S3, S4 and S5 Files). Similar numbers for mappable transcripts were found for tobacco (~18%; ~90%) and rice (~9%; ~94%). On average about 0.7, 1.2, and 1.8% of the reads aligned to *A*. *thaliana*, tobacco and rice, respectively. The above assessment only showed that reads mapped to transcripts, but it is known that some miRNAs can originate from coding sequences and thus these mapped reads could represent miRNAs. Therefore, we assessed the sequence coverage that can be achieved for the detected transcripts (Tables J, N and R in S3, S4 and S5 Files). Here, the sequence coverage is the ratio of nucleotides for which at least one read aligned to the transcript divided by the number of nucleotides in the transcript. Table 3 shows an excerpt for *A*. *thaliana* from the complete data (**Table J in** S3 File) and it is seen that some transcripts have sequence coverage of close to 90%. On average for all samples 2, 2, and 4 transcripts with coverage of over 80% were found for tobacco, *A*. *thaliana*, and rice, respectively (**Tables L, P, and T in** S3, S4 and S5 Files). The distribution for sequence coverage among samples can be seen in Fig 1 and similar figures for the other species are available in S1 File (**Figs A–C in** S1 File). Averaged over all samples, about 1% (*O*. *sativa*), ~2% (*N*. *tabacum*), and ~2% (*A*. *thaliana*) of reads have a higher than 30% sequence coverage (**Tables L, P, and T in** S3, S4 and S5 Files).On average, we found 276 tobacco transcripts in human (682 in porcine), 74 *A*. *thaliana* transcripts in human (1528 porcine), and 347 rice transcripts in human (in porcine 3326) samples. Two human breast milk samples (SRR346518, SRR346519) and one pig sample (SRR445993) contain much less matches to transcripts for all three species (0.1–0.3 fold of remaining samples Tables K, O, and S in S3, S4 and S5 Files).

**Table 3:** Percent read coverage of transcripts from *Arabidopsis thaliana* found in human (first four columns; and last 8 columns for pig). The complete data can be found in Table J in S3 File. This one and similar data for tobacco and rice can be found in Table I in S2 File and their complete data in Tables N and R in S4 and S5 Files, respectively. Very high coverages are bolded. The same data and similar information for *N*. *tabacum* and *O*. *sativa* is also available as Table I in S2 File.

Transcript	SRR346516	SRR346517	SRR346518	SRR346519	SRR445991	SRR445992	SRR445993	SRR445994	SRR445995	SRR445996	SRR445997	SRR445998
**gi|42469794**	**0.89**	**0.82**	0.24	0.08	0.44	0.37	0.36	0.24	0.18	0.14	0.26	**0.51**
**gi|110740128**	**0.63**	**0.72**	0.17	0.15	0.32	0.32	0.29	0.16	0.12	0.20	0.19	0.33
**gi|15810199**	**0.55**	**0.59**	0.20	0.21	0.30	0.33	0.28	0.18	0.13	0.21	0.20	0.34
**gi|20259398**	**0.53**	**0.55**	0.11	0.09	0.14	0.14	0.18	0.07	0.03	0.09	0.12	0.19
**gi|20259359**	**0.51**	**0.59**	0.18	0.07	0.20	0.05	0.14	0.03		0.03	0.07	0.07
**gi|110740643**	0.50	**0.54**	0.33	0.24	0.38	0.46	0.38	0.23	0.20	0.23	0.17	0.22
**gi|20466066**	0.47	**0.61**	0.07	0.09	0.14	0.13	0.09	0.06	0.07	0.11	0.10	0.17
**gi|42468209**	0.45	**0.60**	0.14	0.18	0.17	0.14	0.14	0.05	0.04	0.08	0.11	0.16
**gi|42467781**	0.37	**0.57**	0.07	0.08	0.21	0.25	0.09	0.30	0.05	0.15	0.04	0.25
**gi|116294370**	0.30	0.40	0.07	0.06	0.09	0.09	0.07	0.05	0.03	0.07	0.04	0.16
**gi|42467949**	0.28	0.38	0.03	0.11	0.10	0.06	0.10	0.03	0.03	0.13	0.09	0.13

Only mapped transcripts were used to calculate the distribution (Fig 1; **Table N in** S4 File) and it seems that transcripts had little coverage on average. This could be attributed to an overall low number of reads of the contamination. Interestingly, some showed very high coverage similar to the results for *A*. *thaliana* (Table 3). Similar figures for *O*. *sativa* (**Fig C in** S1 File) and *A*. *thaliana* (**Fig A in** S1 File) are available and the underlying data is presented in Tables R and J in S5 and S3 Files, respectively.

High sequence coverage of up to 94% for foreign transcripts are not expected to be found in breast milk except for from the microbiome and species specific ones. For all samples several transcripts showed higher than 80% sequence coverage, which is enormous considering that only few reads are actually contaminating the respective samples. Three samples show less alignment and lower sequence coverage overall, but email inquiries to determine why remained unanswered. From these results, we conclude that mRNAs must have contaminated the samples, which does not exclude that miRNAs, which may originate from any part of the genome [17], are also among the contaminants or stem from some of the mRNAs. However, it seemed clear from these results that miRNAs were not enriched in breast milk.

### Some Transcripts are Shared Among Samples

**Fig 2:**
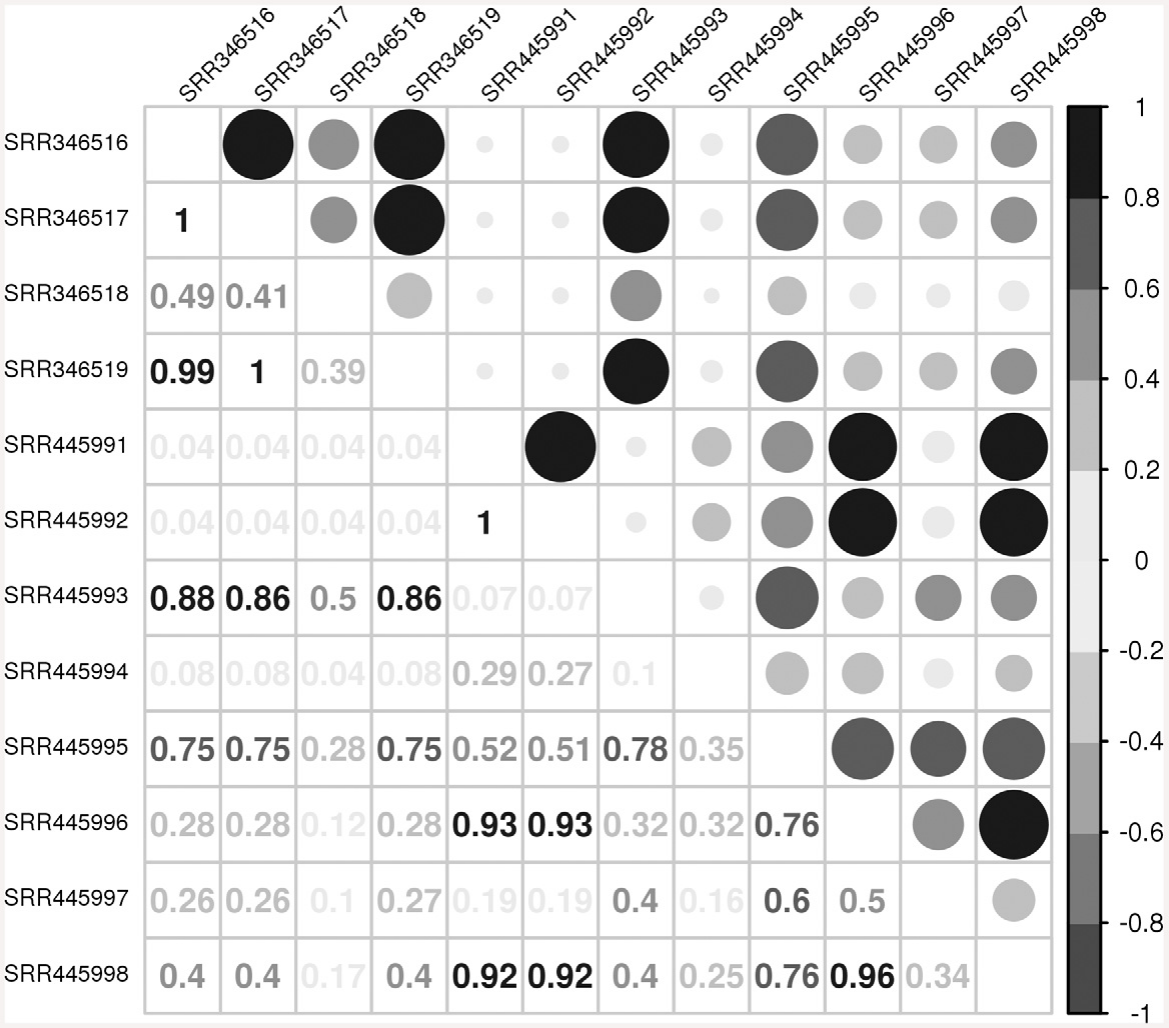
Correlation of Transcripts Between Samples. Correlation among transcripts of *Oryza sativa* found in human and porcine samples. The first 4 samples across the top and the first 4 rows from top correspond to human samples; the remainder is of pig origin. High correlation is visualized via darker color and larger circles. Corresponding figures for *A*. *thaliana* (**Fig D in** S1 File) and *N*. *tabacum* (**Fig E in** S1 File).

After identifying reads mapping to mRNAs in the breast milk samples, we were interested in whether the same mRNAs would be shared among human (4), porcine (8), or all (12) samples. For *A*. *thaliana* about 200 transcripts were detected in human samples, and out of those 21 (~10%) were shared among all human samples (**Table K in** S3 File). Assuming random drawing, the chance for finding a particular transcript shared among samples is:

$$p_{shared}=\frac{n_{1}}{N}\cdot \frac{n_{2}}{N}\cdot \frac{n_{3}}{N}\cdot \frac{n_{X}}{N}$$

where *n*_*x*_ is the number of transcripts found for the sample *x* and *N* is the number of available transcripts. For *A*. *thaliana* the probability that human, pig, or all samples share a particular transcript is 7*10^−9^, 1*10^−6^, and 8*10^−15^, respectively. For rice this is quite similar with 2*10^−8^, 3*10^−6^, and 7*10^−14^, but for tobacco the probability is slightly higher with 3*10^−5^, 1*10^−6^, and 5*10^−11^ since there are less available transcripts. Despite the low probabilities, *A*. *thaliana* and tobacco have 10 transcripts that are shared among all 12 samples while rice has 4 (**Tables K, O and S in** S3, S4 and S5 Files). This calculation was only for one mRNA, but sets of mRNAs can be shared among samples, as well, which can be analyzed using Pearson correlation (Fig 2). A higher correlation means more mRNAs are shared between the samples. Fig 2 provides an example correlation for rice and similar figures for *A*. *thaliana* and tobacco are available in S1 File (**Figs D–F in** S1 File). It is interesting to note that high correlations are not only found between samples from the same species, but also between organisms. For example, the number of mapped *A*. *thaliana* transcripts shared between sample SSR346517 (human) and SRR445993 (pig) is 27. In the Materials and Methods Section we show how to calculate the probability for shared transcripts among samples and conclude that it is extremely low (5*10^−15^). Tables M, Q, and U in S3, S4 and S5 Files show the number of shared transcripts between samples and provide the probability for that to be by chance.

Finding mRNAs in the breast milk samples is problematic for the hypothesis that foodborne xeno-miRNAs, performing cross-kingdom regulations, are deliberately enriched therein. Additionally, we analyzed whether the mRNAs that were identified in the different samples are shared among them. Despite the low probability for that to be possible, we found high correlations between samples (intra and inter-species). Also, if the mRNAs were foodborne, we would expect significant differences at least among the human samples. Finally, we would expect no significant correlation between human and porcine samples, but for many of the samples we found high correlations (Fig 2, **Figs B and C in** S1 File). A practical explanation for this is that the RNAs were contaminations which were introduced after sampling.

### All Identified Organisms were Handled in the Same Institute

The previous sections have detailed how we identified RNAs in the samples and determined that they were potentially contaminants. We, therefore, wondered whether there was any evidence elucidating how these plant RNAs can contaminate the human and porcine breast milk samples. All samples were measured at the Sichuan Agricultural University, Sichuan, China and we inquired via email whether such contaminations were possible, but failed to receive an answer. So we turned to Thomson Reuters’ Web of Knowledge (WOK) to investigate whether any study in respect to the possible contaminating organisms was published during the time period of 2010–2013. If published during this period, the samples for those publications could have been processed in the same lab at the same time as the human and porcine samples. We found some evidence and many of the organisms reported to contribute RNAs to the samples were covered, but not all. Therefore we turned to Google Scholar, which covers more journals than the WOK and found publications for all species during the selected time period (Table 4).

**Table 4:** Hits on Google Scholar when searching for the name of the institute that performed NGS limited to the time period of 2010–2013 and requiring an additional match to the species that were reported to contribute miRNAs to the samples. We reason that a study published in this time period could have been handled at the same time as the human and porcine breast milk samples. The number of Google Scholar hits that contain sequencing or NGS in title or abstract is indicated in parentheses. Please note, however, that contaminants can be introduced during any step of the experimental procedure and not just during sequencing and, therefore, the hits on Google Scholar are the most indicative measure for this analysis. Data is also available as Table B in S2 File.

Popular Plant Name	Plant Species	Porcine Samples	Human Samples	Hits on Google Scholar	Query results limited to the time period 2010–2013
Rice	*Oryza sativa*	Found	Found	**1430 (164)**	"sichuan agricultural university" "Oryza sativa"
Corn	*Zea mays*	Found	Found	**1170 (115)**	"sichuan agricultural university" "zea mays"
Bread wheat	*Triticum aestivum*	Found		**927 (133)**	"sichuan agricultural university" "Triticum aestivum"
Thale cress	*Arabidopsis thaliana*	Found	Found	**607 (179)**	"sichuan agricultural university" "Arabidopsis thaliana"
Soy bean	*Glycine max*	Found	Found	**482 (42)**	"sichuan agricultural university" "glycine max"
Barley	*Hordeum vulgare*	Found	Found	**316 (61)**	"sichuan agricultural university" "Hordeum vulgare"
Tobacco	*Nicotiana tabacum*	Found		**207 (35)**	"sichuan agricultural university" "Nicotiana tabacum"
Sorghum	*Sorghum bicolor*	Found	Found	**204 (29)**	"sichuan agricultural university" "Sorghum bicolor"
Poplar tree	*Populus euphratica*		Found	**193 (11)**	"sichuan agricultural university" "Populus euphratica"
Vine grape	*Vitis vinifera*	Found		**173 (35)**	"sichuan agricultural university" "Vitis vinifera"
Sugarcane	*Saccharum*	Found		**167 (14)**	"sichuan agricultural university" "Saccharum"
Rice	*Oryza rufipogon*	Found		**132 (8)**	"sichuan agricultural university" "Oryza rufipogon"
Citrus	*Citrus sinensis*		Found	**122 (10)**	"sichuan agricultural university" "Citrus sinensis"
Soy bean	*Glycine soja*	Found	Found	**113 (7)**	"sichuan agricultural university" "Glycine soja"
Pig	*Sus scrofa*	X	Found	**106 (59)**	"sichuan agricultural university" "Sus scrofa"
Human	*Homo sapiens*	Found	X	**85 (49)**	"sichuan agricultural university" "Homo sapiens"
Chicken	*Gallus gallus*	Found	Found	**84 (42)**	"sichuan agricultural university" "Gallus gallus"
Cow	*Bos taurus*	Found	Found	**81 (44)**	"sichuan agricultural university" "Bos taurus"
Switchgrass	*Panicum virgatum*	Found		**68 (5)**	"sichuan agricultural university" "Panicum virgatum"
Zebrafish	*Danio rerio*	Found	Found	**55 (26)**	"sichuan agricultural university" "Danio rerio"
Poplar tree	*Populus trichocarpa*	Found	Found	**46 (19)**	"sichuan agricultural university" "Populus trichocarpa"
Citrus	*Citrus x paradisi x Poncirus trifoliata*		Found	**40 (7)**	"sichuan agricultural university" ("Citrus x paradisi" OR "Poncirus trifoliata")
Purple false brome	*Brachypodium distachyon*		Found	**33 (18)**	"sichuan agricultural university" "Brachypodium distachyon"
Cowpea	*Vigna unguiculata*	Found	Found	**32 (2)**	"sichuan agricultural university" "Vigna unguiculata"
European spruce tree	*Picea abies*	Found	Found	**24 (4)**	"sichuan agricultural university" "Picea abies"
Moss	*Physcomitrella patens*		Found	**19 (9)**	"sichuan agricultural university" "Physcomitrella patens"
Dog	*Canis familiaris*	Found	Found	**15 (13)**	"sichuan agricultural university" "Canis familiaris"
Runner bean	*Phaseolus coccineus*	Found		**13 (0)**	"sichuan agricultural university" "Phaseolus coccineus"
Poplar tree	*Populus tremuloides*	Found		**11 (1)**	"sichuan agricultural university" "Populus tremuloides"
Field penny-cress	*Thlaspi arvense*	Found		**10 (0)**	"sichuan agricultural university" "Thlaspi arvense"
Cacao tree	*Theobroma cacao*		Found	**9 (6)**	"sichuan agricultural university" "Theobroma cacao"
Arabidopsis lyrata	*Arabidopsis lyrata*		Found	**4 (4)**	"sichuan agricultural university" "Arabidopsis lyrata"
Colorado Blue Columbine	*Aquilegia coerulea*		Found	**1 (0)**	"sichuan agricultural university" "Aquilegia coerulea"
Orange	*Citrus trifoliata*	Found	Found	**1 (1)**	"sichuan agricultural university" "Citrus trifoliata"

Table 4 summarizes the plant species previously studied and the animal species we specifically added to the analysis. We observed a compound correlation among the number of the studies found for a species, the number of miRNAs known for a species, and the amount of miRNAs found in human and porcine samples for that species (Table 5 **and Table D in** S2 File).

**Table 5:** Comparison of the number of unique miRNAs found in different samples, the number of known miRNAs for that species and the number of publications in respect to that species during the time period 2010–2013 at the institute where measurements were performed. Data is also available as Table G in S2 File.

Species	Popular name	Number of unique miRNAs detected in samples	Number of miRNAs in PMRD or miRBase	% of known miRNAs in PMRD or miRBase	Google scholar hits
*Homo sapiens*	Human	1062	1881	40.37	85
*Bos taurus*	Cow	394	808	17.34	81
*Canis familiaris*	Dog	262	502	10.77	15
*Sus scrofa*	Pig	229	382	8.20	106
*Gallus gallus*	Chicken	227	740	15.88	84
*Danio rerio*	Zebra fish	209	346	7.43	55
*Oryza sativa*	Rice	27	2773	25.45	1430
*Populus trichocarpa*	Poplar tree	25	2780	25.51	46
*Zea mays*	Corn	24	269	2.47	1170
*Arabidopsis thaliana*	Thale cress	12	1530	14.04	607
*Arabidopsis lyrata*	Arabidopsis lyrata	10	375	3.44	4
*Vigna unguiculata*	Cowpea	9	93	0.85	32
*Picea abies*	European spruce tree	9	41	0.38	24
*Sorghum bicolor*	Sorghum	8	173	1.59	204
*Glycine max*	Soy	8	282	2.59	482
*Brachypodium distachyon*	Purple false brome	6	140	1.28	33
*Triticum aestivum*	Bread wheat	5	85	0.78	927
*Physcomitrella patens*	Moss	4	282	2.59	19
*Glycine soja*	Soy	4	15	0.14	113
*Vitis vinifera*	Grape	3	186	1.71	173
*Oryza rufipogon*	Rice	3	6	0.06	132
*Citrus trifoliata*	Citrus	3	6	0.06	1
*Thlaspi arvense*	Field penny-cress	2	1	0.01	10
*Saccharum*	Sugarcane	2	20	0.18	167
*Populus tremuloides*	Poplar tree	2	7	0.06	11
*Panicum virgatum*	Switchgrass	2	1	0.01	68
*Hordeum vulgare*	Barley	2	23	0.21	316
*Citrus x paradisi x Poncirus trifoliata*	Citrus	2	4	0.04	40
*Theobroma cacao*	Cacao tree	1	82	0.75	9
*Populus euphratica*	Poplar tree	1	8	0.07	193
*Phaseolus coccineus*	Runner bean	1	2	0.02	13
*Nicotiana tabacum*	Tobacco	1	1	0.01	207
*Citrus sinensis*	Orange	1	64	0.59	122
*Aquilegia coerulea*	Colorado blue Columbine	1	45	0.41	1

The rows highlighted in gray (Table 5), were added by us to the analysis and we did not perform clustering of reads, which is the reason why their counts cannot be compared to the counts for the plants below and why they are generally larger. Also, animal miRNAs were established using data from miRBase whereas plant miRNAs were established using data from PMRD [16]. Species with many published studies (high likelihood of concurrent handling) and many known miRNAs (high likelihood for spurious assignment) tend to be higher in the list and have more hits in the investigated samples. There are outliers which may possibly be due to concurrent handling with the human and porcine samples. The largest amount of contamination in the porcine (and likely also in the human) samples stems from human (**Table F in** S2 File), which is expected [11]. In other carefully executed studies we generally find between 0.1 and 1% human contamination in plant-derived NGS reads. We tried to find organisms which were not studied at the Sichuan Agricultural University to have potential negative data for our analysis, but failed to do so since any measurement is likely contaminated with human reads which are similar to porcine reads and since any available examples may again be contaminated by other organisms studied at the same lab. For *Syntrichia ruralis*, we could not find any evidence that it was studied at the Sichuan Agricultural University and while we could still map reads to its transcripts, the amount was far less than for all other organisms in this study with the exception of accession SRR346519 (**Table F in** S2 File).

In summary, it can be seen that all organisms, for which Lukasik and Zielenkiewicz reported miRNAs in breast milk, were handled at the same institute which performed the measurements they used and it is, therefore, highly likely that the samples were contaminated in the process.

## Conclusions

In order to assess whether the study by Lukasik and Zielenkiewicz [12] was able to identify cross-kingdom regulation via xeno-miRNAs we considered the following points:

1. Do the xeno-miRNAs actually originate from food sources?
2. The difficulty for xeno-miRNAs to be enriched in breast milk
3. Whether complete mRNAs are found in the samples
4. Whether all reported plant species may have been handled concurrently in the same laboratory during the time period of measurement
5. Whether the number of available miRNAs in miRBase or PMRD correlates with the number of miRNAs found in the samples
6. Whether there is a strong correlation among the RNAs found in human and pig samples even for tobacco which has been only reported to be found in pig, previously
7. The probability of finding sets of RNAs shared among samples
8. That the plant miRNA pathway may have evolved independently [18]
9. The small titer of miRNAs in breast milk

Every point listed above contributes to our overall assessment that the authors of “*In silico* identification of plant miRNAs in mammalian breast milk exosomes—a small step forward?” in-deed identified RNAs in breast milk. In the light of our findings, we believe that their conclusion that these RNAs are foodborne miRNAs which alter gene regulation may not hold. Considering the points above and performing a comprehensive analysis, we argue, presenting evidence, that the RNAs present in the analyzed samples are contaminants. The studies by Zhou et al. [13] and Gu et al. [14] are unaffected by these contamination due to their design and purpose. However, the data from their studies were not suitable for the study attempted by Lukasik and Zielenkiewicz, which prompted us to answer their question with “one step forward, two steps back”. We believe that when analyzing data from public sources special care should be used by taking two steps back and considering all possible sources of errors and contaminations. In conclusion, we suggest that for this type of data analysis best practices should be established, agreed upon, and enforced by journals accepting such studies.

As a final note, we would like to point out, that our findings only question the study by Lukasik and Zielenkiewicz, but not the possible (although, in our opinion, improbable) existence of foodborne miRNAs regulating human gene expression. While we would be very excited about further investigations into cross-kingdom regulation via xeno-miRNAs, we are very reserved about the viability of this process.

## Materials and Methods

### Breast Milk Next Generation Sequencing Datasets

We used 4 human and 8 porcine publicly available breast milk exosome sequencing datasets. Both human and porcine samples were handled at the Sichuan Agricultural University; one tested the enrichment of immune-related microRNAs in human breast milk [13] and the other analyzed lactation-related microRNAs in porcine breast milk [14]. SRA accessions for each dataset and GEO accessions for relevant studies are given in Table E in S2 File. The 4 human samples initially had 31.323.775, 29.656.785, 78.36.132, and 17.557.335 next generation sequencing reads of length 40 and the 8 porcine samples initially had 20.468.495, 24.189.635, 18.224.844, 21.123.540, 23.984.540, 14.590.531, 25.074.431, and 28.141.425 reads of length 40 (**Table F in** S2 File).

### Preprocessing of Next Generation Sequencing Data

We performed quality checks on each dataset by using FastQC [19]. In all samples, many over-represented sequences and low per base sequence quality scores (S1 Fig) showed us that quality trimming was essential before any further analysis. We used an in-house script to detect the over-represented sequences and determined the adapters used in sequencing runs. Then, we employed cutadapt [20] to trim reads from adapters and remove low quality regions (at a quality threshold of 30) or to discard reads if they are mostly adapters or of low quality (at a length threshold of 17). Discarding by adapter contaminations and low quality reads left 24.870.393, 22.977.089, 2.638.515, and 6.746.662 reads in human and 3.170.143, 4.528.620, 2.980.639, 1.799.177, 711.125, 2.554.206, 1.151.433, and 3.446.872 reads in porcine samples (**Table F in** S2 File).

In order to eliminate reads of human and pig origin, the remaining reads were mapped to their respective genomes (hg19 for human, Sscrofa9.53 for pig) by bowtie (version 1.1.1) [21], allowing 1 mismatch in the seed region (-n switch). In the following, we treated reads that were not aligned to their respective genomes as foreign nucleic acid sequences, either stemming from contaminations or coming from other sources, such as foodborne microRNAs.

### Mining Organisms Studied at the Sichuan Agricultural University

In order to investigate whether organisms detected in the samples that were analyzed at the Sichuan Agricultural University, Sichuan, China at a time when they could have contaminated the samples studied here, we queried Google Scholar. First we extracted all plant species that were identified by Lukasik and Zielenkiewicz from their S1 and S2 Files S1 [12] which lead to our Table 1. We restricted the Google Scholar (http://scholar.google.com) search for publications during the time period from 2010–2013 (**Table D in** S2 File). We reason, that the published datasets could have been measured at any time during this period and thus any other plant that was analyzed during that time was a potential source of contamination. With these settings we searched for “Sichuan Agricultural University” and each identified organism enclosed in quotation marks individually (Table 4).

### Mapping NGS Reads to Transcripts from Selected Plant Species

We aligned reads, which were not mapped to their respective genomes, to the coding sequences or the transcripts of three selected plant species in the same manner they were aligned to genomes (see above). We used *Arabidopsis thaliana* transcripts from TAIR (version 20101108) [22], *Nicotiana tabacum* transcripts from NCBI (date: 06.11.2014), and *Oryza sativa* transcripts from PlantGDB (version 193) [23] for the mapping (**Table F in** S2 File).

To calculate sequence coverage for transcripts, we counted the number of nucleotides in each transcript that was mapped by at least one read, and divided that by the transcript length.

### Analysis of Identified Transcripts Shared Among Samples

**Fig 3:**
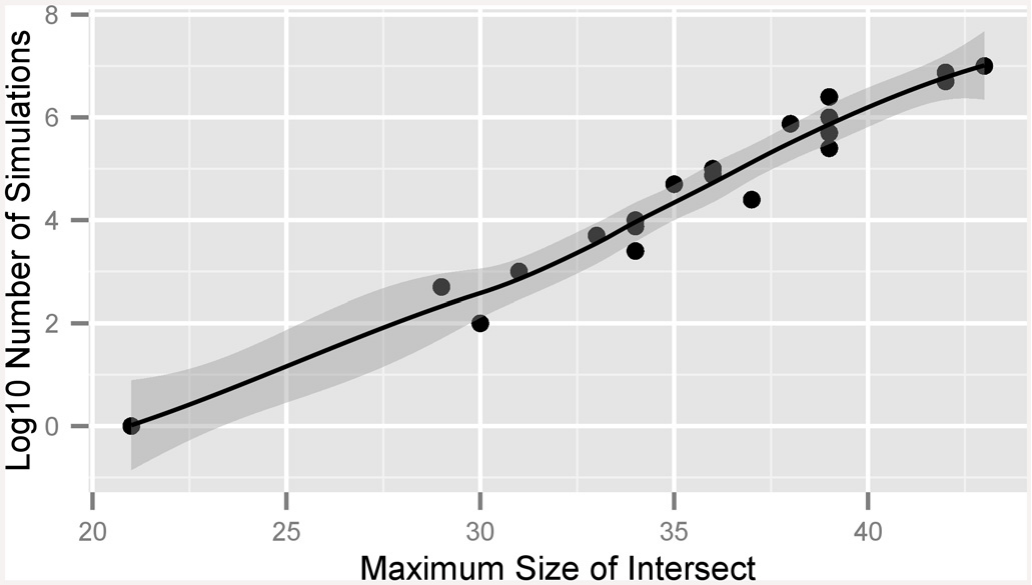
Simulation to Estimate Probability of Shared Transcripts. Simulation results for estimating the likelihood of finding shared transcripts in independent samples. The number of simulations necessary to generate at least one time a shared set of the given size is given as black dots. Dark gray indicates the confidence interval for the fitted curve.

We simulated the probability of finding multiple shared transcripts in both human and porcine samples under the assumption of randomness (Fig 3). For this, we created a set of numbers from 1 to 80.000, and created two subsets from the universal set by randomly drawing numbers without replacement of sizes 200 and 7.000. We then calculated the sizes of intersections between the small and large subsets at each iteration and took the maximum of them. We calculated the maximum length of subset for 20 different numbers of simulations from 1 to 10.000.000.

From the simulation we arrived at a mathematical description for the probability that *x* number of transcripts were shared between two samples.

$$P\left(x\right)=\frac{\left(C\left(\begin{array}{c} N-a\\ b-x \end{array}\right)\cdot C\left(\begin{array}{c} a\\ x \end{array}\right)\right)}{C\left(\begin{array}{c} N\\ b \end{array}\right)}$$

Where *N* is the universal set, *a* one set of transcripts (*a* < *b*), and *b* the other set (*b* > *a*), *x* is their intersection (*a* ∩ *b*), and *P*(*x*) the probability of finding *x* shared elements between them. It should be noted that we assumed random drawing, while it is not exactly applicable since some transcripts appear more often than others. We believe that this influence on the outcome is rather low since we abstract from this problem by looking only at distinct transcripts that have been identified in at least one sample and also do not factor in their abundance.

### Correlation of Transcripts Identified in Human and Porcine Samples

To investigate the degree of similarity, the identified sets of transcripts and their read counts were between all samples, we calculated pairwise Pearson-correlation values for the three selected plant species by using the corrplot package in R (https://github.com/taiyun/corrplot). We excluded transcripts that were not identified in any sample and worked with only those that were found at least once in one sample.

### Identification of Animal Transcripts in Human and Porcine Samples

We chose two animal food-sources (chicken, cow) and two that are not common food-sources (zebrafish, dog) to test whether their miRNAs can be detected in human and porcine breast milk samples. We acquired mature miRNA sequences from miRBase and also included human miRNAs when testing against porcine samples and porcine miRNAs when testing against human samples. We created a BLAST [24] databases from human and porcine reads that were not aligned to their genomes, and used 'blastn' in 'blastn-short' task to search mature miRNA sequences in reads. We then counted the number of NGS reads each miRNA was mapped to. We did not perform any read clustering in this study, thus the read numbers we calculated contained duplicates and were larger than those calculated by Lukasik and Zielenkiewicz for plant species.

## Supporting Information

S1 Fig Sequencing quality of the samples analyzed in this study. (TIFF)

S1 File Contains Figures A–F. Distribution of transcript coverage for *Arabidopsis thaliana* in human and porcine samples (**Figure A**). Distribution of transcript coverage for *Nicotiana tabacum* in human and porcine samples (**Figure B**). Distribution of transcript coverage for *Oryza sativa* in human and porcine samples (**Figure C**). Correlation among transcripts of *Arabidopsis thaliana* found in human and porcine samples (**Figure D**). Correlation among transcripts of *Nicotiana tabacum* found in human and porcine samples (**Figure E**). Corre lation among transcripts of *Oryza sativa* found in human and porcine samples (**Figure F**).
(DOCX)

S2 File Contains Supplementary Tables A–I. Assessment of whether identified species are food sources (**Table A**). Query results from Google Scholar in respect to whether organisms were analyzed at the Sichuan Agricultural University (**Table B**). Number of miRNAs that we detected in the breast milk samples from selected animals (**Table C**). Integrated table showing the detection status in human and porcine breast milk samples and the number of uniquely detected miRNAs (**Table D**). Information about human and porcine breast milk samples (**Table E**). Read mapping statistics for human and porcine breast milk samples (**Table F**). Relationship among uniquely identified miRNAs, number of available miRNAs in PMRD and miRBase and number of studies that investigated an organism at the Sichuan Agricultural University (**Table G**). Correlation of *A*. *thaliana*, tobacco, and rice miRNAs shared between samples (**Table H**). Messenger RNA coverage by reads for transcripts from Tobacco, *A*. *thaliana*, and rice (**Table I**).
(XLSX)

S3 File Contains Supplementary Tables J–M. Sequence coverage of *A*. *thaliana* transcripts by reads from human and porcine breast milk samples (**Table J**). Count of *A*. *thaliana* transcripts that were identified with at least one read in human and porcine breast milk samples (**Table K**). Distribution of *A*. *thaliana* transcript coverage for the individual samples (**Table L**). Number of shared *A*. *thaliana* transcripts identified in multiple samples and the associated probability (**Table M**).
(XLSX)

S4 File Contains Supplementary Tables N–Q. Sequence coverage of *N*. *tabacum* transcripts by reads from human and porcine breast milk samples (**Table N**). Count of *N*. *tabacum* transcripts that were identified with at least one read in human and porcine breast milk samples (**Table O**). Distribution of *N*. *tabacum* transcript coverage for the individual samples (**Table P**). Number of shared *N*. *tabacum* transcripts identified in multiple samples and the associated probability (**Table Q**).
(XLSX)

S5 File Contains Supplementary Tables 18–21. Sequence coverage of *O*. *sativa* transcripts by reads from human and porcine breast milk samples (**Table R**). Count of *O*. *sativa* transcripts that were identified with at least one read in human and porcine breast milk samples (**Table S**). Distribution of *O*. *sativa* transcript coverage for the individual samples (**Table T**). Number of shared *O*. *sativa* transcripts identified in multiple samples and the associated probability (**Table U**).
(XLSX)
