# Supplementary figures and images for: One Step Forward, Two Steps Back; Xeno-MicroRNAs Reported in Breast Milk Are Artifacts
Source: PLoS One. 2016 Jan 29;11(1):e0145065. doi: 10.1371/journal.pone.0145065 (PMC4732600; doi:10.1371/journal.pone.0145065)

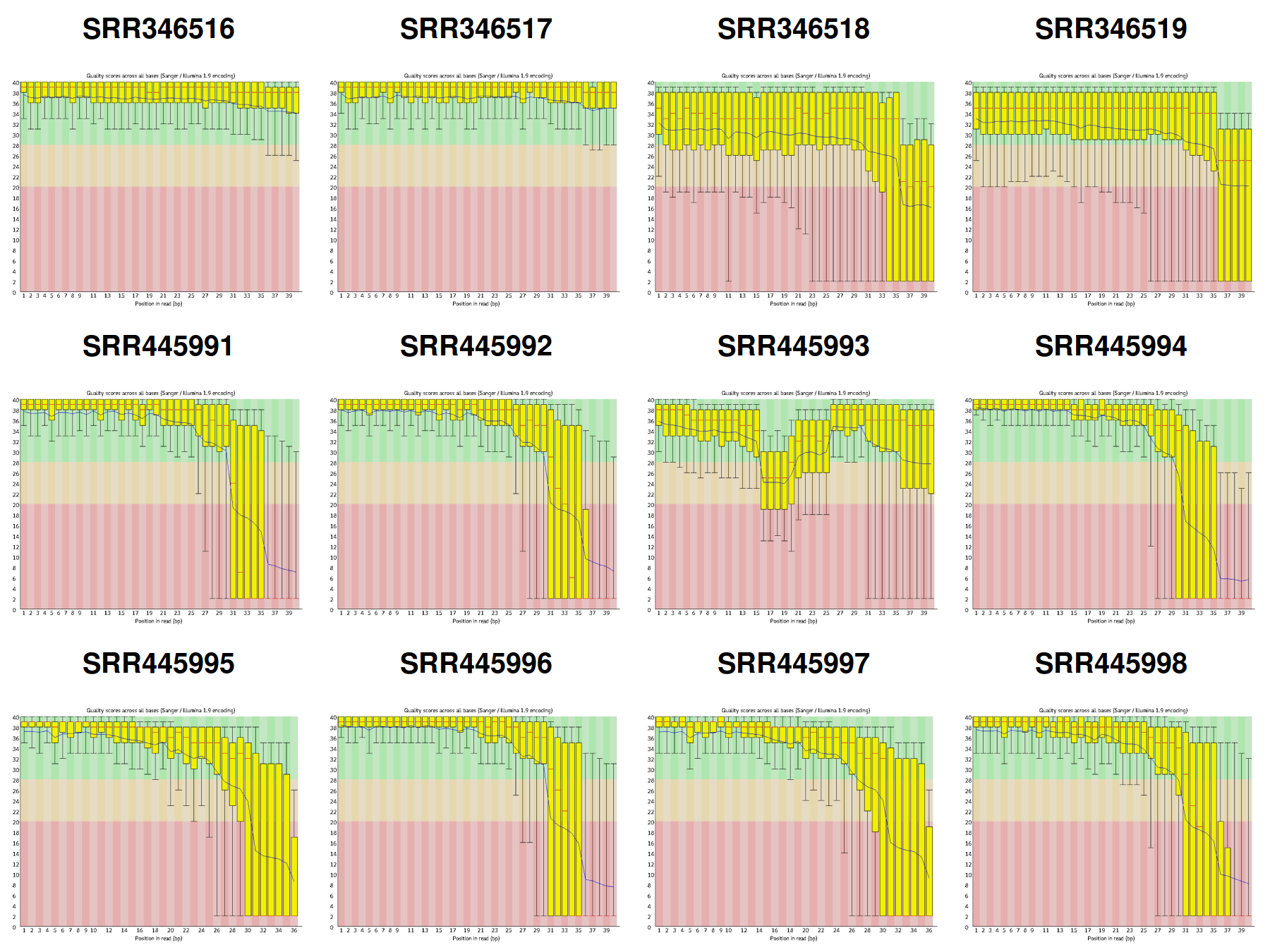

Supplement: S1 Fig — (TIFF) [file pone.0145065.s001.tiff]
