# Supplementary material for: One Step Forward, Two Steps Back; Xeno-MicroRNAs Reported in Breast Milk Are Artifacts
Source: PLoS One. 2016 Jan 29;11(1):e0145065. doi: 10.1371/journal.pone.0145065 (PMC4732600; doi:10.1371/journal.pone.0145065)
Supplement: S1 File — Distribution of transcript coverage for Arabidopsis thaliana in human and porcine samples (Figure A). Distribution of transcript coverage for Nicotiana tabacum in human and porcine samples (Figure B). Distribution of transcript coverage for Oryza sativa in human and porcine samples (Figure C). Correlation among transcripts of Arabidopsis thaliana found in human and porcine samples (Figure D). Correlation among transcripts of Nicotiana tabacum found in human and porcine samples (Figure E). Corre lation among transcripts of Oryza sativa found in human and porcine samples (Figure F). (DOCX) [file pone.0145065.s002.docx]

One Step Forward, Two Steps Back; Xeno-MicroRNAs Reported in Breast Milk are Artifacts

Caner Bağcı^1^ and Jens Allmer^2,3,*^

^1^Biotechnology, Izmir Institute of Technology, Urla, Izmir, Turkey

^2^Molecular Biology and Genetics, Izmir Institute of Technology, Urla, Izmir, Turkey

^3^Bionia Incorporated, IZTEKGEB A8, Urla, Izmir, Turkey

# Abstract

**Background**

MicroRNAs (miRNAs) are short RNA sequences that guide post-transcriptional regulation of gene expression via complementarity to their target mRNAs. Discovered only recently, miRNAs have drawn a lot of attention. Multiple protein complexes interact to first cleave a hairpin from nascent RNA, export it into the cytosol, trim its loop, and incorporate it into the RISC complex which is important for binding its target mRNA. This process works within one cell, but circulating miRNAs have been described suggesting a role in cell-cell communication.

**Controversy**

Viruses and intracellular parasites like *Toxoplasma gondii* use miRNAs to manipulate host gene expression from within the cellular environment. However, recent research has claimed that a rice miRNA may regulate human gene expression. Despite ongoing debates about these findings and general reluctance to accept them, a recent report claimed that foodborne plant miRNAs pass through the digestive tract, travel through blood to be incorporated by alveolar cells excreting milk. The miRNAs are then said to some immune-related function in the newborn.

**Principal Findings**

We acquired the data that supports their claim and performed further analyses. In addition to the reported miRNAs, we were able to detect almost complete mRNAs and found that the foreign RNA expression profiles among samples are exceedingly similar. Inspecting the source of the data helped understand how RNAs could contaminate the samples.

**Conclusion**

Viewing these findings in context with the difficulties foreign RNAs face on their route into breast milk and the fact that many identified foodborne miRNAs are not from actual food sources, we can conclude beyond reasonable doubt that the original claims and evidence presented may be due to artifacts. We report that the study claiming their existence is more likely to have detected RNA contamination than miRNAs.

# Transcript Coverage


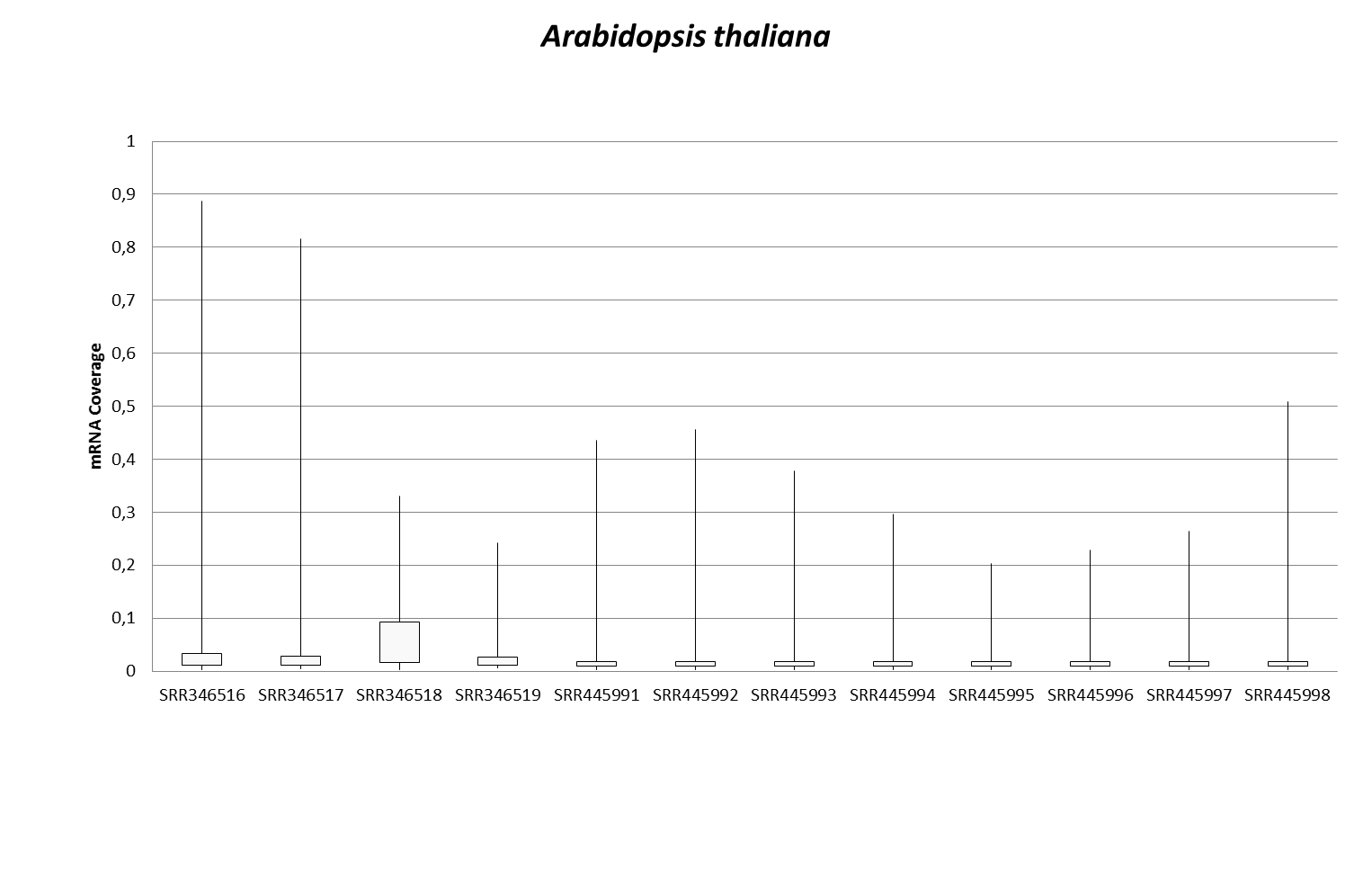


**Figure A:** Distribution of transcript coverage for *Arabidopsis thaliana* in human (first four) and porcine (last 8) samples. Data can be found in Table J in File S3.


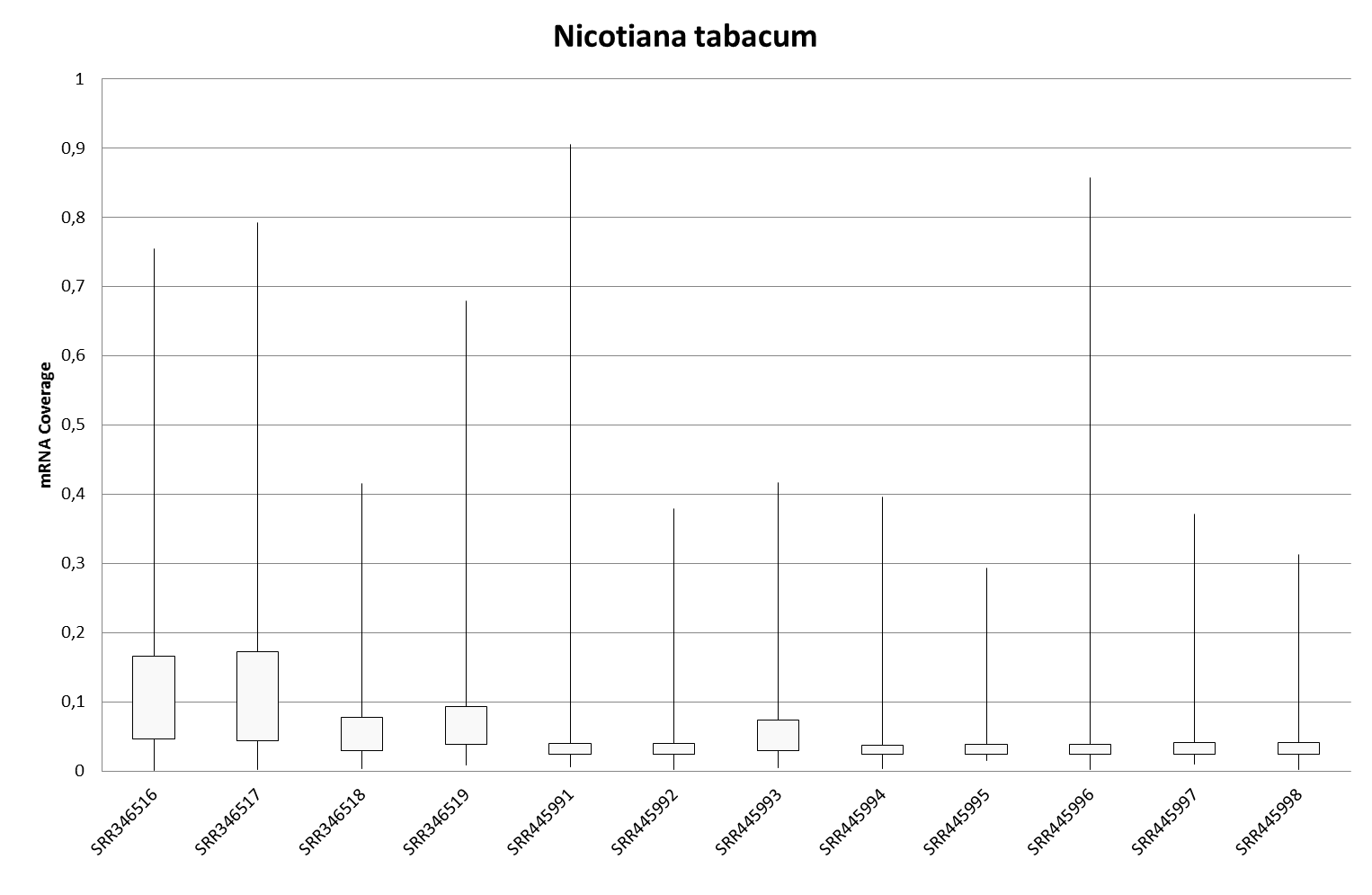


**Figure B:** Distribution of transcript coverage for *Nicotiana tabacum* in human (first four) and porcine (last 8) samples. Data can be found in Table N in File S4.


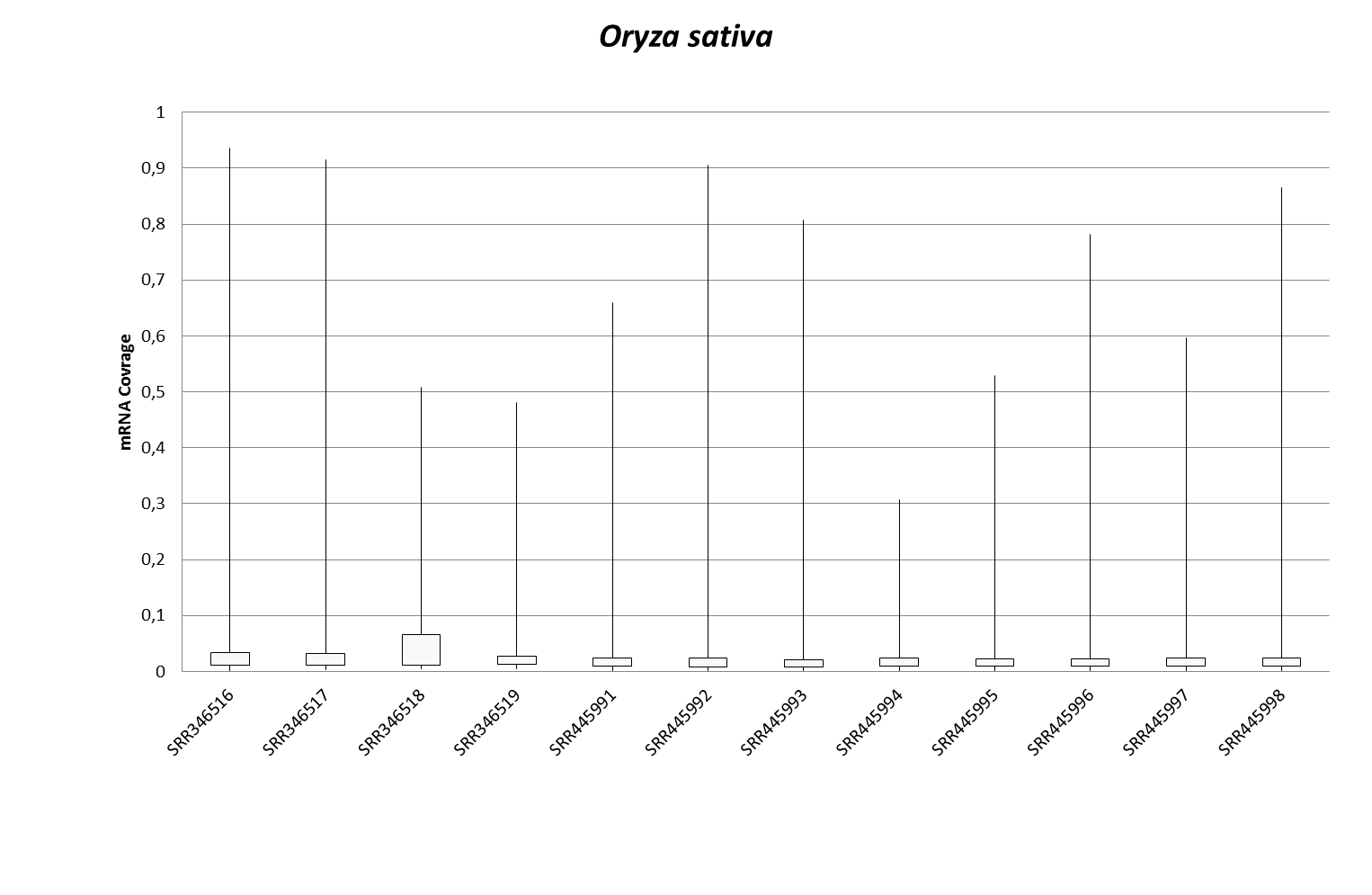


**Figure C:** Distribution of transcript coverage for *Oryza sativa* in human (first four) and porcine (last 8) samples. Data can be found in Table R in File S5.


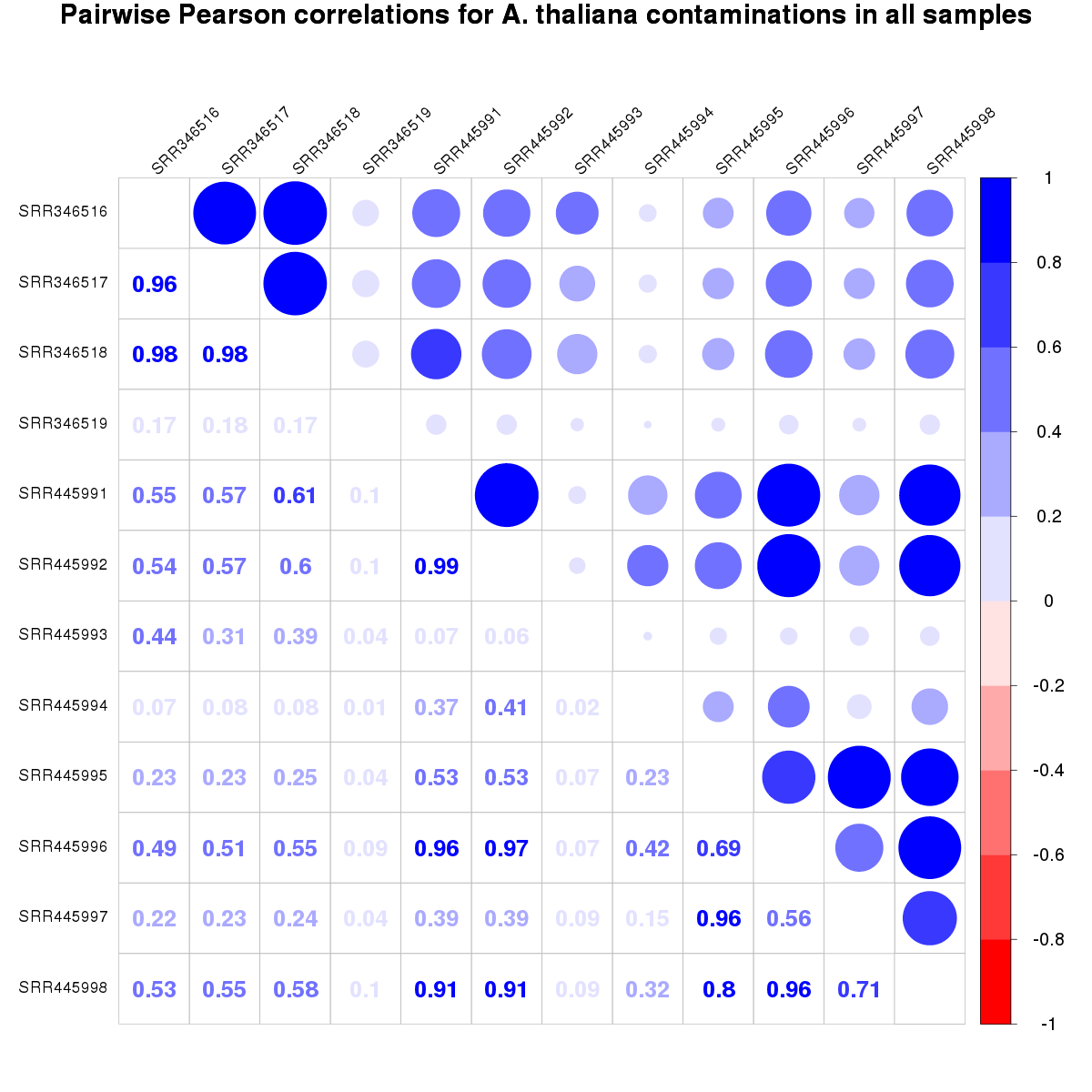


**Figure D:** Correlation among transcripts of *Arabidopsis thaliana* found in human and porcine samples. The first four samples across the top and the first four rows from top correspond to human samples; the remainder is of pig origin. High correlation is visualized via darker color and larger circles.


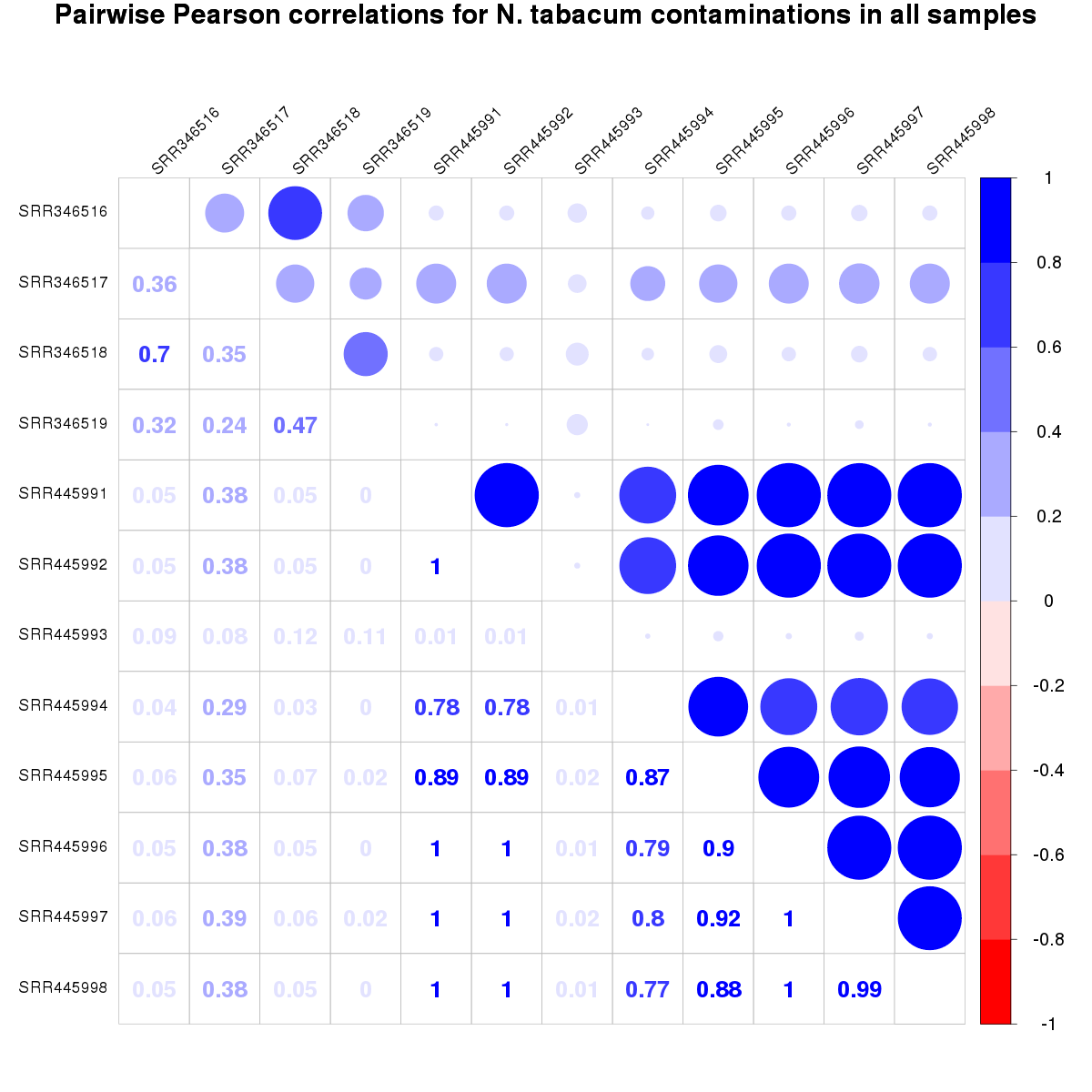


**Figure E:** Correlation among transcripts of *Nicotiana tabacum* found in human and porcine samples. The first four samples across the top and the first four rows from top correspond to human samples; the remainder is of pig origin. High correlation is visualized via darker color and larger circles.


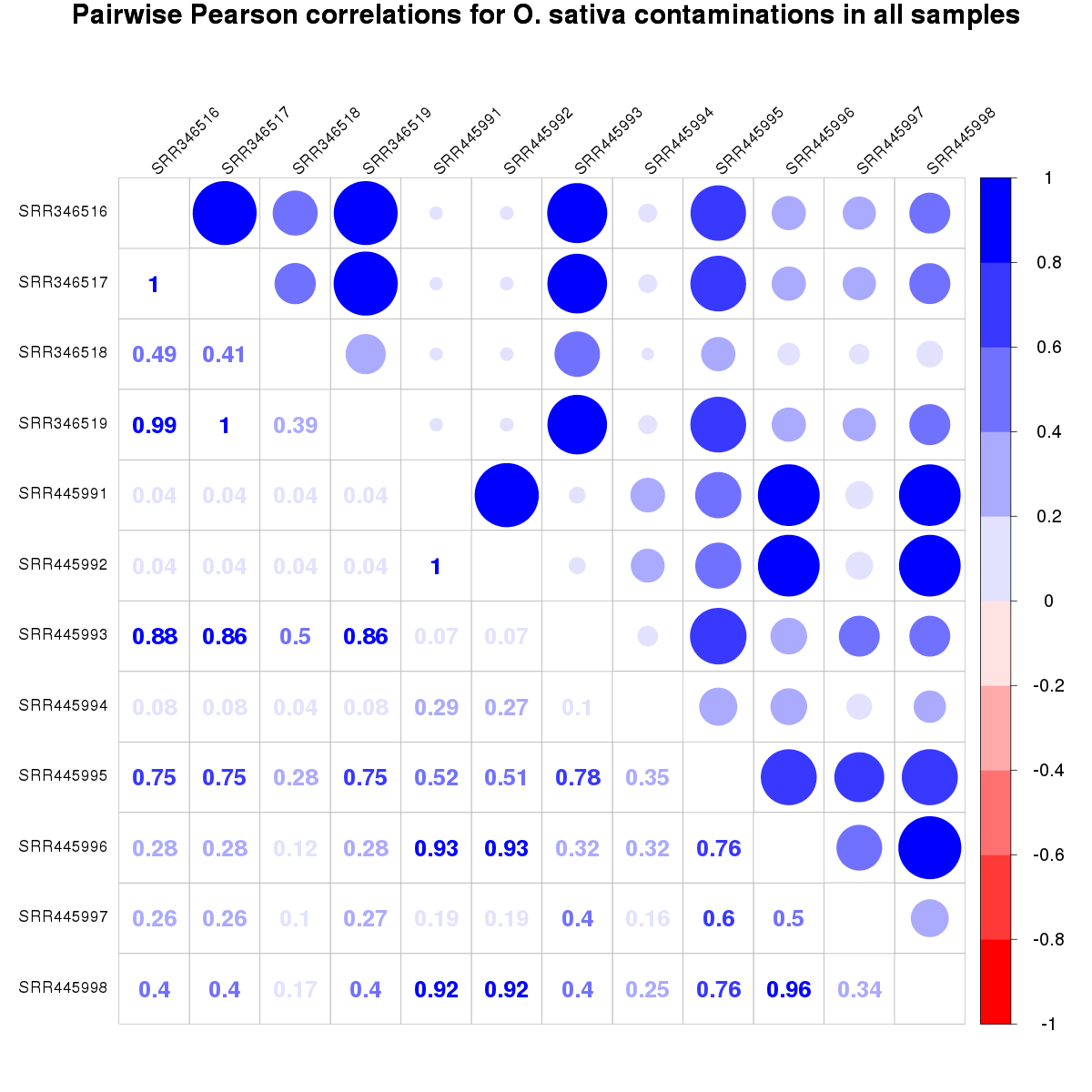


**Figure F:** Correlation among transcripts of *Oryza sativa* found in human and porcine samples. The first four samples across the top and the first four rows from top correspond to human samples; the remainder is of pig origin. High correlation is visualized via darker color and larger circles.
